# Single oral administration of dronabinol increases ocular blood flow in patients with glaucoma

**DOI:** 10.1111/aos.17573

**Published:** 2025-08-07

**Authors:** Theresa Lindner, Viktoria Pai, Patrick Janku, Nikolaus Hommer, Anton Hommer, Marihan Abensperg‐Traun, Liudmyla Petric, Leopold Schmetterer, Gerhard Garhöfer, Doreen Schmidl

**Affiliations:** ^1^ Department of Clinical Pharmacology Medical University of Vienna Vienna Austria; ^2^ VIROS – Vienna Institute for Research in Ocular Surgery – Karl Landsteiner Institute, Hanusch Hospital Vienna Austria; ^3^ Hommer Ophthalmology Institute Vienna Austria; ^4^ Department of Child and Adolescent Psychiatry Medical University of Vienna Vienna Austria; ^5^ Singapore Eye Research Institute, Singapore National Eye Centre Singapore Singapore; ^6^ Ophthalmology and Visual Sciences Academic Clinical Program, Duke‐NUS Medical School Singapore Singapore; ^7^ SERI‐NTU Advanced Ocular Engineering (STANCE), Nanyang Technological University Singapore Singapore; ^8^ School of Chemistry, Chemical Engineering and Biotechnology Nanyang Technological University Singapore Singapore; ^9^ Center for Medical Physics and Biomedical Engineering Medical University of Vienna Vienna Austria; ^10^ Fondation Ophtalmologique Adolphe De Rothschild Paris France; ^11^ AIER Eye Hospital Group Changsha China

**Keywords:** cannabinoids, dronabinol–tetrahydrocannabinol, glaucoma, laser speckle flowgraphy, optic nerve head blood flow, optical coherence tomography angiography, randomized controlled clinical trial, vessel densities

## Abstract

**Purpose:**

Glaucoma is a leading cause of irreversible blindness globally, primarily driven by elevated intraocular pressure (IOP). Still, some patients progress despite significant IOP lowering, potentially due to impaired ocular blood flow. This study aimed to evaluate the effects of dronabinol, a synthetic tetrahydrocannabinol derivative, on ocular blood flow in primary open‐angle glaucoma (POAG) patients.

**Methods:**

This randomized, double‐masked, placebo‐controlled, cross‐over study included 23 patients with treated POAG (mean age 68 ± 7 years). All participants received dronabinol (11 patients received 5 mg and 12 received 10 mg in a randomized fashion) on one study day and placebo on the other study day. The primary outcome was optic nerve head blood flow (ONHBF) measured by laser speckle flowgraphy. Mean blur rate was determined for the large vessel area (MV), the tissue area (MT) and the total ONH area (MA). Secondary outcomes included vessel densities assessed by optical coherence tomography angiography, IOP, and blood pressure.

**Results:**

Administration of 10 mg dronabinol significantly increased ONHBF (MA: 10.8 ± 20.6%, *p* = 0.018, MV: 12.0 ± 24.8%, *p* = 0.042, and MT: 11.0 ± 22.6%, *p* = 0.022, each vs. placebo) up to 4 h post‐administration without affecting IOP or mean arterial pressure (*p* > 0.548 each). Additionally, a significant increase in vessel density in the superficial vascular plexus was found after administration of 10 mg dronabinol (6.7 ± 14.7%, *p* = 0.040 vs. 5 mg).

**Conclusion:**

This pilot study demonstrates that systemic dronabinol enhances ONHBF in glaucoma patients, suggesting its potential as adjunct therapy for glaucoma by targeting vascular dysfunction. Further longitudinal studies are needed to explore its long‐term impact on disease progression and visual field preservation.

**Trial Registration:**

ClinicalTrials.gov ID: NCT04596826.

## 1 INTRODUCTION

Glaucoma is the major cause of irreversible blindness worldwide, with elevated intraocular pressure (IOP) being the main risk factor for the onset and progression of the disease (Jayaram et al., 2023). Lowering IOP still remains the only proven treatment option available (Spaeth, 2021). While this therapeutic approach is effective for the majority of patients, a considerable number of patients continue to experience disease progression despite significant IOP lowering (Tribble et al., 2023).

There is substantial evidence that ocular blood flow is significantly altered in glaucoma patients (Alasbali, 2023; Harris et al., 2020; Kiyota et al., 2021; Tobe et al., 2015). Studies have consistently demonstrated that retinal and optic nerve head (ONH) blood flow is reduced in individuals with glaucoma compared with healthy controls (Bata et al., 2019; Garhöfer et al., 2022). Furthermore, this reduction in blood flow has been shown to correlate with the severity of the disease, suggesting that vascular dysfunction might play a critical role in glaucoma pathophysiology (Calzetti et al., 2022; Kuroda et al., 2020; Orgül et al., 2020). Currently, no approved pharmacological treatments are available to directly improve ocular blood flow.

Tetrahydrocannabinol (THC) has long been considered a treatment option for glaucoma, mainly because of its IOP‐lowering properties (Joshi et al., 2024; Lindner et al., 2023). In addition, THC may also increase ocular blood flow, at least in healthy subjects (Hill et al., 2020; Lindner et al., 2023; Plange et al., 2007). Recent data from our laboratory confirm that even low doses of THC, which neither affect IOP nor induce systemic psychoactive side effects, can increase ocular blood flow considerably (Hommer et al., 2020, 2021). This increase in blood flow is likely mediated by the activation of CB_1_ receptors, which promote vasorelaxation through inhibition of Endothelin‐1 (ET‐1) and activation of the nitric oxide (NO) system (Alonso & Radomski, 2003; Lindner et al., 2023; Ronco et al., 2007; Stanley & O'Sullivan, 2014). Both systems have been suggested to play a significant role in ocular blood flow and its regulation, as well as in glaucoma pathophysiology (Erdinest et al., 2021; Lommatzsch et al., 2022; Resch et al., 2009; Schmidl et al., 2011; Toda & Nakanishi‐Toda, 2007).

Currently, data on the effect of THC on ocular blood flow in glaucoma patients is still lacking. The present pilot study set out to investigate the effect of a single systemic dose of dronabinol administered in two different dosages on ocular blood flow in glaucoma patients. Dronabinol, a synthetic tetrahydrocannabinol derivate ((−)‐trans‐Δ^9^‐Tetrahydrocannabinol), is a registered medication that is legally available in most European countries and the United States of America for treatment of several conditions, although it is not currently indicated for glaucoma. The study was conducted in a randomized, placebo‐controlled, double‐masked, cross‐over design.

## 2 MATERIALS AND METHODS

Between November 2020 and June 2024, glaucoma patients were enrolled at the Department of Clinical Pharmacology, Medical University of Vienna. The study was conducted in accordance with the Declaration of Helsinki and followed the guidelines for Good Clinical Practice of the European Union. The study protocol was approved by the Ethics Committee of the Medical University of Vienna and the national competent authorities. All participants provided written informed consent prior to any trial‐related activity. Sample size calculation is based on previous measurements of optic nerve head blood flow (ONHBF) using laser speckle flowgraphy (LSFG) in our laboratory. Given the variability in our previous experiments (standard deviation of approximately 13%), an alpha error of 0.05 and a power of 0.80, this sample size calculation aimed to detect changes in ONHBF of 10% (Witkowska et al., 2017). Changes in ONHBF less than 10% were considered to be irrelevant.

### 2.1 In/exclusion criteria

Glaucoma patients were included if they had a diagnosis of a manifest primary open‐angle glaucoma, defined as pathological optic disc appearance, glaucoma hemifield test outside normal limits (but mean deviation [MD] < 10 dB) and/or untreated IOP ≥ 21 mmHg on at least three measurements in the medical history. Further inclusion criteria were age ≥ 18 years, ametropia ≤ 6 dioptres, non‐smokers, normal findings (or an abnormality deemed clinically irrelevant by the investigator) in the medical history and physical examination including electrocardiogram (ECG) and laboratory testing. Main exclusion criteria were other types of glaucoma, filtration surgery at any time or a laser procedure for glaucoma within the last 12 months. Other exclusion criteria included ocular inflammation or infection within the last 3 months, intraocular surgery within the last 6 months, medication or clinical condition that potentially could interact with dronabinol, abuse of alcoholic beverages or drugs, relevant psychiatric disorders and risk for drug dependence evaluated by a psychiatrist, pregnancy, blood donation and clinically relevant illness in the 3 weeks before the study.

### 2.2 Experimental design

The present study was conducted in a monocentre, double‐masked, randomized, placebo‐controlled, cross‐over design. Randomization lists were generated by a member of the Department of Clinical Pharmacology not involved in the study procedures using a computer software (http://www.randomization.com) with the method of randomly permuted blocks. A randomization envelope was prepared for each subject in accordance with the randomization list.

Each participant underwent a screening examination for eligibility in the 4 weeks prior to the first study day, which included an automated 30–2 visual field testing (if not done in the six previous months and/or results not available) and a retinal nerve fibre layer thickness (RNFLT) assessment using the Heidelberg Spectralis Optical Coherence Tomography (OCT) system (Heidelberg Engineering, Germany).

Two identical study days were scheduled, during which each subject received dronabinol (5 mg or 10 mg) or placebo on the first study day and vice versa on the second study day, with a washout period of at least 3 weeks in between. Twelve hours before the study days, the subjects had to abstain from alcohol and stimulating beverages containing xanthine derivatives. On the study days, the subjects arrived after a light meal and sleep for 7–8 hours at the study centre. First, a pregnancy test in females of childbearing potential, a urine drug screening test as well as an alcohol breath test were performed. Afterwards, one drop of tropicamide 0.5% (Mydriaticum “Agepha” 0.5%, Austria) was instilled in the study eye and a resting period of 20 min was scheduled to ensure constant haemodynamic conditions. Thereafter systemic haemodynamics (in particular systolic, diastolic, mean arterial pressure and pulse rate) and IOP were measured. Ocular perfusion pressure (OPP) was calculated from mean arterial pressure (MAP) and IOP as 2/3 MAP – IOP (Leske, 2009; Robinson et al., 1986). A 10°x10° macular volume scan using the Heidelberg Spectralis OCT Angiography (OCT‐A) module assessed the vessel densities in different slabs. ONHBF was measured by LSFG (Nidek, Japan). At the end of the measurement period, patients were randomly assigned to receive dronabinol (5 mg or 10 mg) or placebo. After a 1‐hour resting period, the aforementioned haemodynamic, IOP, OCT‐A, and LSFG measurements were repeated. The measurements were started 1 hour after administration, since maximum plasma levels of THC are usually reached 60–120 min after administration (Grotenhermen, 2003; Plange et al., 2007). LSFG measurements were further repeated 4 and 6 h (±30 min) after dronabinol intake to determine prolonged effects. All subjects remained in the hospital for at least 6 h after study drug administration. A follow‐up safety visit was performed 7–14 days after the last study day.

### 2.3 Study medication

Capsules used in this investigation contained 5 mg dronabinol (Bionorica ethics, Neumarkt, Germany), also referred to as (−)‐trans‐Δ^9^‐THC. The 5 mg dronabinol group received one capsule containing 5 mg dronabinol and one capsule placebo. The 10 mg dronabinol group received two capsules containing 5 mg dronabinol (total dose of 10 mg). Placebo capsules were identical in appearance to the dronabinol capsules with no active ingredient. On every study day, patients received two capsules together with 15 g butter, two pieces of bread, and 250 mL of water for comparable bioavailability. Dronabinol and placebo capsules were provided by Allerheiligen Apotheke, Mag. Pharm. Herbert Baldia KG, Vienna under Good Manufacturing Practice conditions.

### 2.4 Methods

#### 2.4.1 Optical coherence tomography angiography (OCT‐A)

For capillary‐specific analysis, 10° × 10° (3 × 3 mm^2^) macula‐centred high‐resolution (512 B‐scans, 512 A‐scans/B‐scan) OCT‐A‐scans were performed. Vessel density indices of the superficial vascular plexus (SVP), the intermediate capillary plexus (ICP), and the deep capillary plexus (DCP) were analysed from the acquired data sets. These three different OCT‐A slabs were generated per subject per timepoint based on the plexus architecture of Campbell et al. (Campbell et al., 2017) using the standard slab settings of the built‐in Heidelberg OCT‐A software (Heidelberg Eye Explorer version 1.10.2.0). After a quality check, the raw en face scans of SVP, ICP, and DCP were loaded into a custom MATLAB program (MathWorks, Natick, MA, version R2023b). Images were binarized by thresholding at the mean intensity of the respective images to obtain the corresponding vessel density indices. For calculation of SVP, large vessels were isolated on SVP scans using a fixed threshold (intensity > 130).

#### 2.4.2 Laser Speckle Flowgraphy (LSFG)

In the present study, the same settings were used for blood flow measurements before and after dronabinol administration (1, 4 and 6 h). Data evaluation was performed with the LSFG analysis software (LSFG Analyser, Version 1.0.1.1). The ONH area was marked manually by positioning an ellipsoid region of interest at the ONH margin. All images were checked for quality, and any artefacts not detected by the analysing software were removed manually using the Multi View module. The mean blur rate (MBR) in arbitrary units (a.u.) was determined for the large vessel area (MV, “mean MBR of vascular area”), the tissue area (MT, “mean MBR of tissue area”) and the total ONH area (referred to as MA, “mean MBR of all area” and referring to the composite of MV and MT). To discriminate between visible surface vessels and ONH tissue areas the threshold for the MBR signal intensity was automatically calculated using histogram analysis.

### 2.5 Statistical analysis

Statistical analysis was performed using IBM SPSS Statistics (Version 28, IBM, Armonk, NY, USA). All values are presented as means ± SD. The Kolmogorov–Smirnov test was used to confirm normal distribution for all major outcome variables. Descriptive statistics are reported for all values obtained. Percent changes between baseline values and after drug administration were calculated for the main outcome variables. To compare the effect between the three groups (placebo, 5 and 10 mg dronabinol), a repeated‐measures ANOVA model was used. Within the model, planned contrasts were used to detect differences in the changes in outcome parameters between placebo and treatment groups (5 and 10 mg). A p‐value <0.05 was considered the level of significance.

Due to insufficient image quality, two glaucoma patients were excluded from the analysis of LSFG parameters and four glaucoma patients from OCT‐A analysis of vessel densities.

For outcome variables, per‐protocol analysis was performed. For Adverse Event evaluation, the whole data set, including drop‐outs, was used.

Figures were created using Graph Pad Prism (Version 10.3.0, GraphPad Software, CA, USA).

### 2.6 General description of the study population

**FIGURE 1:**
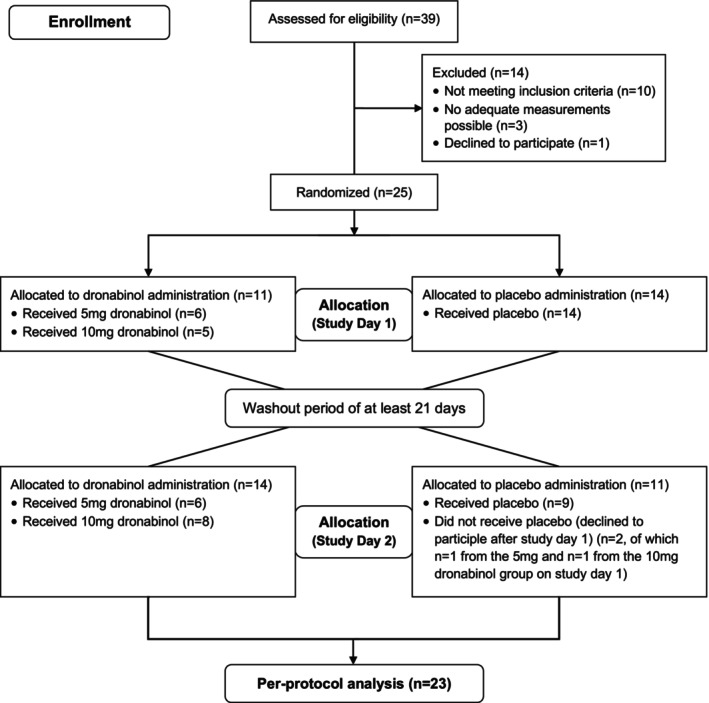
Trial flow diagram.

A total of 25 Caucasian patients with diagnosed and treated primary open‐angle glaucoma were included in the present study, of which 23 finished the study according to the protocol and were included in the per‐protocol analysis. Of these, 11 patients received 5 mg and 12 patients received 10 mg dronabinol in a randomized fashion. Figure 1 shows the accountability of subjects. Two subjects withdrew consent after the first study day due to personal reasons. Mean age was 68 ± 7 years and gender distribution was 10 m:13 f. Baseline values are presented in Table 1.

**TABLE 1:** Baseline data of included glaucoma patients. Data are presented as mean ± SD.

VF mean deviation (dB)	−3.3 ± 3.1
RNFLT (μm)	70.0 ± 11.5
MAP (mmHg)	104 ± 11
IOP (mmHg)	16 ± 3
OPP (mmHg)	53 ± 8
MA (a.u.)	25.9 ± 6.0
MV (a.u.)	49.8 ± 10.6
MT (a.u.)	17.4 ± 4.9
SVP (%)	22.6 ± 1.8
ICP (%)	31.0 ± 2.9
DCP (%)	32.0 ± 2.8

Abbreviations: DCP, vessel density in the deep capillary plexus; ICP, vessel density in the intermediate capillary plexus; IOP, intraocular pressure; MA, mean blood flow in the area of the optic nerve head; MAP, mean arterial pressure; MT, mean tissue blood flow; MV, mean blood flow in large vessels; OPP, ocular perfusion pressure; RNFLT, retinal nerve fibre layer thickness; SVP, vessel density in the superficial vascular plexus; VF, visual field.

The most frequent concomitant diagnoses were arterial hypertension (*n* = 13) and hypercholesterolemia (*n* = 9). Table 2 shows the number of glaucoma medications taken by patients.

**TABLE 2:** Number of prescribed IOP‐lowering medications and previous laser procedures for glaucoma.

Number of medications	No laser procedure	SLT (>12 months ago)	Total
1	8	1	9
2	5	1	6
3	6	0	6
4	2	0	2
Total	21	2	23

Abbreviation: SLT, selective laser trabeculoplasty.

## 3 RESULTS

### 3.1 Effect of dronabinol on ocular blood flow

Dronabinol had no significant effect on IOP, MAP or OPP (*p* > 0.548 each).

**FIGURE 2:**
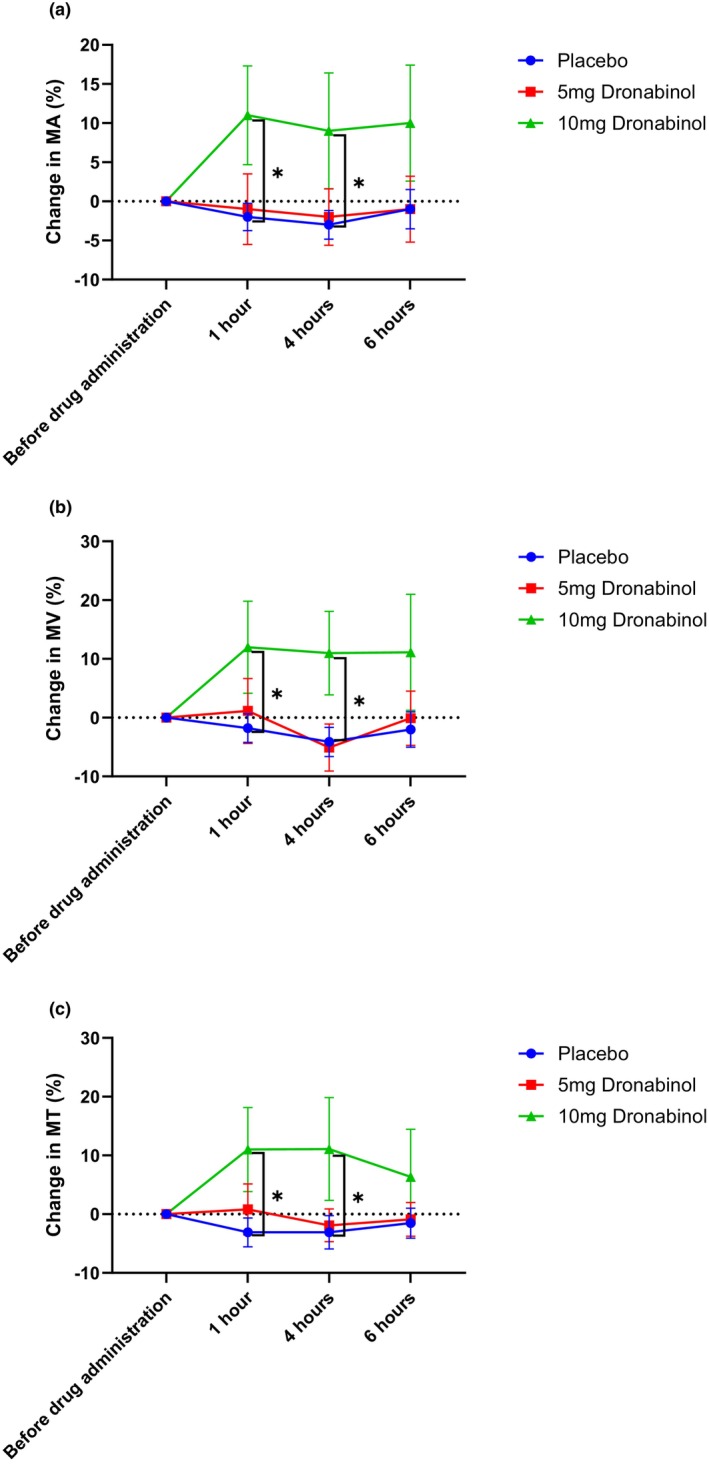
Relative change from baseline in mean blood flow in the area of the optic nerve head (MA, panel a), mean blood flow in large vessels (MV, panel b) and mean tissue blood flow (MT, panel c) after administration of either placebo, 5 mg or 10 mg dronabinol in glaucoma patients. *Significant vs. placebo. Data are presented as mean ± SEM.

For MA, repeated‐measures ANOVA revealed a significant difference in MA among the three groups (*p* = 0.038). Administration of 10 mg dronabinol increased MA from 25.2 ± 5.4 to 27.6 ± 5.9 a.u. (10.8 ± 20.6%, *p* = 0.018 vs. placebo and *p* = 0.052 vs. 5 mg dronabinol) while almost no change was observed following placebo administration or 5 mg dronabinol (−2.2 ± 7.9% and −1.2 ± 15.2%, respectively). This increase induced by 10 mg dronabinol in MA was significant vs. placebo until 4 h after administration. Figure 2a shows the time course of MA after administration of the study medication.

No significant difference between the three groups was seen within the repeated‐measures ANOVA model for MV and MT (*p* = 0.139 and *p* = 0.073). When comparing the 10 mg group with the placebo group, a significant increase in both parameters (from 46.7 ± 9.6 to 51.5 ± 10.8 a.u., 12.0 ± 24.8%, *p* = 0.042 vs. placebo for MV and from 17.5 ± 3.8 to 19.2 ± 4.6 a.u., 11.0 ± 22.6%, *p* = 0.022 vs. placebo for MT) was found after administration which lasted until 4 hours after administration (Figure 2b,c).

**FIGURE 3:**
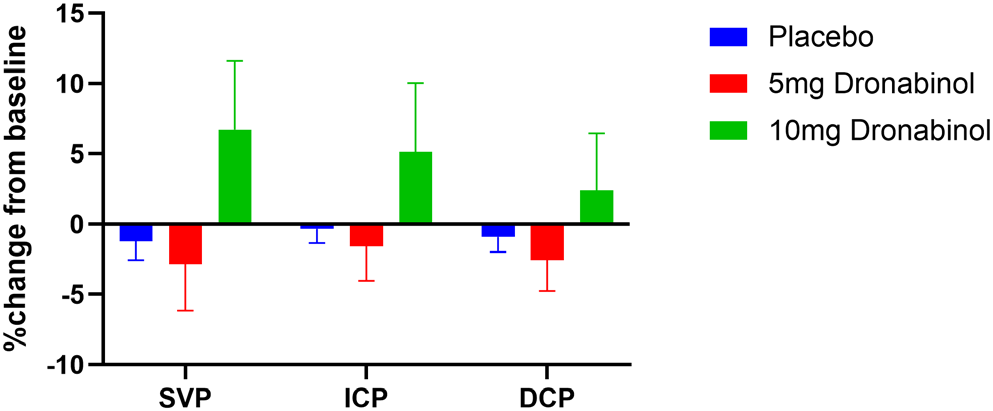
Relative change from baseline in vessel density in the superficial vascular plexus (SVP), intermediate capillary plexus (ICP) and deep capillary plexus (DCP) after drug administration in glaucoma patients.

For vessel densities as assessed with OCT‐A, the ANOVA model showed no significant differences between the three groups (*p* = 0.075 for SVP, *p* = 0.231 for ICP and *p* = 0.404 for DCP). Planned contrasts revealed that the increase in SVP was significantly higher after administration of 10 mg dronabinol compared with 5 mg dronabinol (6.7 ± 14.7%, *p* = 0.040). No significant differences between any of the groups were found for ICP or DCP. Figure 3 shows the relative changes in vessel density after administration of the study medication in glaucoma patients.

An overview of all parameters before and after administration of the study drugs is provided in Table S1.

### 3.2 Adverse events

In total, 9 out of 25 patients experienced an adverse event (AE) during the study that was judged as possibly or probably related to the study drug (Table S2). All patients recovered on the same or the following day, and all AEs were categorized as mild or moderate. No subject dropped out of the study because of an AE. No serious AEs were observed.

## 4 DISCUSSION

This study provides novel insights into the effects of systemic dronabinol on ocular blood flow in glaucoma patients, indicating vasodilator effects even in low doses with no or negligible psychoactive side effects. In particular, our findings demonstrate that a single dose of 10 mg dronabinol significantly increases blood flow in glaucoma patients, with no impact on IOP, MAP, and OPP. This increase was sustained for up to 4 h post‐administration and was reflected in both vessel and tissue measurements.

The vasodilatory effects of cannabinoids have been well‐documented across various vascular regions, including the brain, the heart, and the mesenteric circulation (Járai et al., 1999; Ogunbiyi et al., 2020; Sultan et al., 2018; Wagner et al., 1999). As for the eye, cannabinoids, namely, THC and its synthetic derivative dronabinol, have also demonstrated potential vasoactive effects. Data from Plange et al. showed that oral dronabinol at a dosage of 7.5 mg lowered IOP and was associated with a prolonged arteriovenous transit time, indicating an increase in blood velocity (Plange et al., 2007). In another investigation, the arteriolar and venular diameters of 55 frequent cannabis users were compared with those of 51 control participants using retinal imaging. The latter study demonstrated that on average, cannabis users exhibited larger retinal arteriolar diameters, which again was interpreted as evidence of a retinal vasodilatory effect of cannabinoids (Hill et al., 2020).

More recently, in a study of our laboratory, Hommer et al. (2020) expanded on these findings. In particular, the study showed that orally administered dronabinol at a dose of 5 mg significantly increased ONHBF in healthy subjects without affecting IOP or systemic haemodynamics (Hommer et al., 2020). Additionally, we showed that a single oral administration of 5 mg dronabinol increases retinal blood flow in healthy young subjects (Hommer et al., 2021). In the present study, administration of 5 mg dronabinol had no effect on ocular blood flow, while administration of 10 mg induced a significant increase in ONH blood flow. A reason for this difference could be that the patient cohort was significantly older than the healthy subjects included in the previous studies. Ageing has been found to be associated with changes in cannabinoid receptor binding in various brain regions, including the basal ganglia and hippocampus, particularly for the CB_1_ receptor (Marchalant et al., 2008; Romero et al., 1998). Thus, differences in receptor configuration and/or sensitivity in elderly subjects may account for these differences. Secondly, the endocannabinoid system per se is known to decline with age, showing, for example, lower levels of the major endocannabinoid 2‐arachidonoylglycerol (Nidadavolu et al., 2022). This might lead to altered sensitivity to exogenous cannabinoids like dronabinol. However, a final proof of this hypothesis would require additional data in respect to cannabinoid receptors in glaucoma patients, such as a cross‐sectional study in patients with different severity of glaucoma or a prospective trial in patients with progressive glaucomatous disease, which is currently lacking.

Our findings indicate that in glaucoma patients, cannabinoids exert a vasodilatory and blood flow increasing effect. These results are further supported by the OCT‐A data investigating the microvascular effects of dronabinol. Our analysis revealed a significant increase in vessel density within the SVP following 10 mg dronabinol administration compared with the 5 mg dose. Interestingly, no significant changes were observed in the ICP or DCP. This layer‐specific response pattern suggests that dronabinol's effects may be anatomically selective, potentially due to differences in vascular anatomy, receptor distribution, or local regulatory mechanisms across retinal layers. Notably, our findings are also supported by the dose‐dependent effects of dronabinol, showing consistently superior effects of the 10 mg dosage across multiple parameters. This dose dependency validates the biological plausibility of our findings and suggests a direct pharmacological effect.

A key strength of this trial lies in its rigorous methodology. The double‐masked, randomized, placebo‐controlled, cross‐over design minimizes the risk of bias and ensures the reliability of the findings.

While the study provides valuable insights, certain limitations must be acknowledged. First, approximately half of the study participants had arterial hypertension and were therefore taking vasoactive medications which may impact the results. However, this reflects the typical glaucoma population and therefore the results should be valuable to characterize drug effects in this specific demographic. Second, the study was limited by only testing two doses of dronabinol (5 and 10 mg). While the dose‐dependent response observed suggests potential benefit from higher doses, this must be weighed against the potential corresponding increase in adverse effects. Higher doses would likely amplify adverse effects, potentially outweighing a possible therapeutic benefit. Additionally, as the drug effect was still present at the end of the observation period, the duration of action may be longer than currently documented. Thus, it is possible that the 10 mg dose might already provide a sustained benefit beyond the observation time of 4 hours without requiring dose escalation. This highlights the need for longer duration studies to fully characterize the pharmacodynamic profile. Additionally, it has to be noted that the relatively small sample size, although sufficient to detect changes in blood flow, lacks the statistical power and duration necessary to evaluate potential long‐term clinical benefits such as preservation of visual field. Because of all these limitations, the present study more has the character of a pilot study. As the trial focused on the acute effects of dronabinol, future long‐term studies incorporating larger cohorts, extended treatment durations, and longitudinal assessments of structural, functional, and safety outcomes are needed.

In summary, our study demonstrates that administration of 10 mg dronabinol leads to a significant increase in ocular blood flow in patients with glaucoma. The effect seems to be dose‐dependent, as a single administration of 5 mg had no effect on ocular blood flow. These findings highlight the potential role of dronabinol in improving ocular perfusion in glaucoma patients but also underscore the importance of considering age and disease‐related factors when assessing cannabinoid‐mediated vascular effects. Further investigations with larger sample sizes, longer study durations, and multiple applications instead of single intake are warranted to evaluate the potential clinical benefits of this therapeutic approach.

## FUNDING INFORMATION

This research was funded in whole by the Austrian Science Fund (FWF) KLI 854.

## Supporting information


Table S1.

Table S2.
