# Supplementary material for: Single oral administration of dronabinol increases ocular blood flow in patients with glaucoma
Source: Acta Ophthalmol. 2025 Aug 7;104(2):225–32. doi: 10.1111/aos.17573 (PMC12888942; doi:10.1111/aos.17573)
Supplement: Supplementary file 1 — Table S1. [file AOS-104-225-s001.docx]

|  | **Before drug administration** | | | **After drug administration** | | |
| --- | --- | --- | --- | --- | --- | --- |
|  | **Placebo** | **5mg Dronabinol** | **10mg Dronabinol** | **Placebo** | **5mg Dronabinol** | **10mg Dronabinol** |
| **MAP (mmHg)** | 105 ± 11 | 104 ± 12 | 104 ± 10 | 106 ± 11 | 103 ± 5 | 104 ± 10 |
| **IOP (mmHg)** | 16 ± 3 | 17 ± 4 | 15 ± 3 | 15 ± 3 | 16 ± 3 | 14 ± 3 |
| **OPP (mmHg)** | 54 ± 9 | 52 ± 9 | 54 ± 8 | 55 ± 8 | 52 ± 5 | 55 ± 9 |
| **MA (a.u.)** | 25.8 ± 5.5 | 26.6 ± 6.6 | 25.2 ± 5.4 | 25.2 ± 5.3 | 25.7 ± 5.0 | 27.6 ± 5.9 |
| **MV (a.u.)** | 51.2 ± 10.0 | 52.3 ± 11.2 | 46.7 ± 9.6 | 50.1 ± 10.1 | 51.8 ± 9.3 | 51.5 ± 10.8 |
| **MT (a.u.)** | 17.3 ± 4.4 | 17.0 ± 5.6 | 17.5 ± 3.8 | 16.7 ± 4.5 | 16.7 ± 4.9 | 19.2 ± 4.6 |
| **SVP (%)** | 23.0 ± 1.0 | 23.2 ± 1.9 | 21.8 ± 2.2 | 22.7 ± 1.4 | 22.5 ± 1.9 | 23.1 ± 1.7 |
| **ICP (%)** | 31.5 ± 1.9 | 31.8 ± 1.4 | 30.8 ± 3.3 | 31.3 ± 2.0 | 31.3 ± 2.3 | 31.9 ± 0.9 |
| **DCP (%)** | 32.3 ± 2.2 | 33.2 ± 0.8 | 31.3 ± 3.1 | 32.0 ± 1.7 | 32.3 ± 2.1 | 31.8 ± 1.4 |
