# Supplementary material for: Single oral administration of dronabinol increases ocular blood flow in patients with glaucoma
Source: Acta Ophthalmol. 2025 Aug 7;104(2):225–32. doi: 10.1111/aos.17573 (PMC12888942; doi:10.1111/aos.17573)
Supplement: Supplementary file 2 — Table S2. [file AOS-104-225-s002.docx]

| **Adverse Event** | **Dronabinol** | | **Placebo** | **Total** |
| --- | --- | --- | --- | --- |
|  | **5mg** | **10mg** |  |  |
| Dizziness | 1 | 4 | 0 | 5 |
| Headache | 1 | 1 | 1 | 3 |
| Vomiting | 0 | 2 | 0 | 2 |
| Lightheadedness | 1 | 1 | 0 | 2 |
| Tiredness | 0 | 1 | 0 | 1 |
| Nausea | 0 | 1 | 0 | 1 |
| Syncope | 0 | 1 | 0 | 1 |
| Feeling of weakness | 0 | 1 | 0 | 1 |
| Total | 3 | 12 | 1 | 16 |
